# Metabolic and hormonal effects of ‘catch‐up’ sleep in men with chronic, repetitive, lifestyle‐driven sleep restriction

**DOI:** 10.1111/cen.12747

**Published:** 2015-03-06

**Authors:** Roo Killick, Camilla M. Hoyos, Kerri L. Melehan, George C. Dungan, Jonathon Poh, Peter Y. Liu

**Affiliations:** ^1^ NHMRC Centre for Integrated Research and Understanding of SleepWoolcock Institute of Medical ResearchUniversity of SydneySydneyNSWAustralia; ^2^ Faculty of MedicineUniversity of SydneySydneyNSWAustralia; ^3^ Los Angeles Biomedical Research Institute at Harbor‐UCLA Medical Center and David Geffen School of MedicineUniversity of California Los AngelesTorranceCAUSA

## Abstract

**Objective:**

Acutely restricting sleep worsens insulin sensitivity in healthy individuals whose usual sleep is normal in duration and pattern. The effect of recovery or weekend ‘catch‐up’ sleep on insulin sensitivity and metabolically active hormones in individuals with chronic sleep restriction who regularly ‘catch‐up’ on sleep at weekends is as yet unstudied.

**Design:**

19 men (mean ± SEM age 28·6 ± 2·0 years, BMI 26·0 ± 0·8 kg/m^2^) with at least 6 months’ history (5·1 ± 0·9 years) of lifestyle‐driven, restricted sleep during the working week (373 ± 6·6 min/night) with regular weekend ‘catch‐up’ sleep (weekend sleep extension 37·4 ± 2·3%) completed an in‐laboratory, randomized, crossover study comprising two of three conditions, stratified by age. Conditions were 3 weekend nights of 10 hours, 6 hours or 10 hours time‐in‐bed with slow wave sleep (SWS) suppression using targeted acoustic stimuli.

**Measurements:**

Insulin sensitivity was measured in the morning following the 3rd intervention night by minimal modelling of 19 samples collected during a 2‐h oral glucose tolerance test. Glucose, insulin, c‐peptide, leptin, peptide YY (PYY), ghrelin, cortisol, testosterone and luteinizing hormone (LH) were measured from daily fasting blood samples; HOMA‐IR, HOMA‐β and QUICKI were calculated.

**Results:**

Insulin sensitivity was higher following three nights of sleep extension compared to sustained sleep restriction. Fasting insulin, c‐peptide, HOMA‐IR, HOMA‐β, leptin and PYY decreased with ‘catch‐up’ sleep, QUICKI and testosterone increased, while morning cortisol and LH did not change. Targeted acoustic stimuli reduced SWS by 23%, but did not alter insulin sensitivity.

**Conclusions:**

Three nights of ‘catch‐up’ sleep improved insulin sensitivity in men with chronic, repetitive sleep restriction. Methods to improve metabolic health by optimizing sleep are plausible.

## Introduction

Chronic, lifestyle‐driven sleep restriction is common in many modern ‘24/7’ societies, with about 40% of individuals relying on discretional time on weekends to ‘catch‐up’ on sleep curtailment during the working week.^1^, ^2^ The prevalence of obesity and type 2 diabetes mellitus is increasing to epidemic proportions, particularly in developing nations, in line with increasing globalization, changes in nutrition and sedentary lifestyles.^3^ Epidemiological, interventional and molecular experiments provide a strong rationale linking sleep restriction with these metabolic disorders. Recent large epidemiological studies have associated sleep loss to the development of both obesity^4^ and diabetes mellitus,^1^ and short sleep duration to increased subcutaneous fat.^5^ Experimentally restricting or perturbing sleep for 1–14 nights in duration worsens insulin sensitivity in healthy individuals whose usual sleep is normal in duration and pattern.^1^ Molecular experiments show that adipocytes from sleep‐restricted individuals are resistant to insulin's effects on phosphorylated Akt, a mediator in the insulin‐signalling pathway.^6^ Together, these data indicate that acute sleep restriction is metabolically harmful.

Although 40% of individuals ‘catch‐up’ on sleep over the weekend, the metabolic effects of catch‐up sleep are relatively understudied with no interventional studies to date. Cross‐sectional epidemiological studies in children show that weekend ‘catch‐up’ sleep is associated with a decreased risk of being overweight compared to perpetual short sleepers.^7^, ^8^, ^9^ In adults, an hour of weekend ‘catch‐up’ sleep was associated with a 39% decreased risk of hypertension.^10^ Given these epidemiological data, we therefore examined whether three nights of a saturating amount of ‘catch‐up’ sleep following regular weekday sleep curtailment would improve insulin sensitivity in those with a history of such sleep patterns, compared to sustained sleep restriction. We also tried to unravel mechanisms. An exploratory aim was to examine the effect of targeted acoustic perturbation of slow‐wave sleep (SWS) on insulin sensitivity as SWS has been implicated mechanistically in glucose homoeostasis.^11^ Finally, we also explored the effect of both sleep restriction and experimental perturbation of SWS on other hormones known to modify insulin sensitivity and food intake.

## Methods

### Study protocol

The study complied with Good Clinical Practice guidelines, applicable regulatory requirements and the Declaration of Helsinki. All participants provided written informed consent to participate in the study, which was approved by the Sydney South West Area Health Service Human Research and Ethics Committee (Concord Zone). The study is registered with the Australia New Zealand Clinical Trials Network, www.anzctr.org.au, number ACTRN12609000123246.

### Screening and participants

Healthy male subjects aged between 18 and 50 years were recruited through local advertising. Inclusion criteria included regular sleep–wake patterns as per the description below and being agreeable to spend two weekends at the research institute. Exclusions included shift workers, habitual napping (more than once per month from history), diabetes mellitus, a history of, or symptoms suggesting, a co‐existing sleep disorder, including insomnia, obstructive sleep apnoea, parasomnias or restless legs syndrome. Those with uncontrolled medical conditions or a history of psychiatric disorders or drug abuse, including use of any sedative or neuroactive medications, or indeed any medication that might affect sleep, were also excluded. Subjects could not have crossed time zones within 1 month of the study visits.

Screening included a full medical history, physical examination and detailed explanation of the study protocol. No subject had type 2 diabetes mellitus from history, confirmed by oral glucose tolerance test. Habitual sleep–wake patterns were objectively assessed over 2 weeks with at‐home actigraphy incorporating sleep diary verification of sleep onset and wake‐up times (Actiwatch^™^, Philips/Respironics, Murrysville, PA, USA), analysed by two investigators. Subjects were included if mean weekday nightly sleep period over 2 weeks, between Monday and Thursday nights inclusive, was less than 6·5 h/night and mean nightly weekend sleep period, Friday and Saturday nights, was greater than 25% of the weekday mean. Sleep‐disordered breathing was excluded by three nights' assessment with a portable single‐channel nasal flow recording device (Flow Wizard^™^, DiagnoseIT, Sydney, Australia).^12^

### Randomization

**Figure 1:**
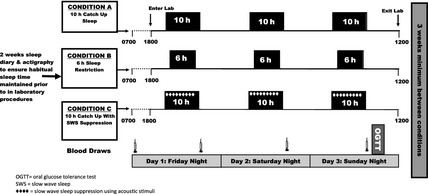
Study design. Subjects were randomized to undergo two of three (young men) or two (older men) conditions in random order: that is, AB, BA, AC, CA, BC or CB in young men; AB or BA in older men. There were 3 weeks washout between conditions.

All participants underwent two out of two or three potential study conditions, in a randomized order, two‐period crossover design. The three potential study conditions were 3 weekend nights (Friday night to Monday morning) of (A) 10 h time in bed (TIB) each night, (B) 6 h TIB each night or (C) 10 h TIB with SWS suppression by acoustic stimuli (10 h↓SWS) each night (Fig. 1). Those aged ≤35 years (group 1) could be randomized to any two of the three conditions. Those >35 years (group 2) could only be randomized to Condition A (6 h TIB) or Condition B (10 h TIB). Men >35 years were not randomized to Condition C (10 h↓SWS) because SWS is already reduced in this age group. Two separate randomization lists for young and older men were computer‐generated in blocks of 4. There was a minimum of 3 weeks of washout between each study visit.

### Study visits

#### General

For 2 weeks prior to a study weekend visit, subjects were asked to maintain their regular ‘catch‐up’ sleep‐wake schedules at home and this was verified by inspection of actigraphy and sleep diaries, with any deviation resulting in a study weekend being rescheduled. Subjects were asked to restrict caffeine and alcohol to two or less drinks or units per day at home. The study was conducted within the chronobiology laboratory in the research institute. Subjects were encouraged to be sedentary and not to sleep outside of scheduled times, and had their own bedroom with ensuite with access to a shared living area. Ambient lighting was kept at less than 50 lux for the duration of the study visit to minimize any phase shift. Subjects were not permitted to exercise or leave the chronobiology laboratory. Study staff ensured subjects did not nap, through continuous camera or direct visual surveillance.

#### Sleep scheduling

Time of lights out was calculated by the subject's screening actigraphy. The weekday (Monday to Thursday nights inclusive) mean sleep centre‐point for each subject was calculated, and lights‐on and lights‐off times were individually centred on that time for each condition. Subjects were only told of their lights‐off time immediately prior to bed on the first evening. They were instructed that if they woke prior to lights on, they should remain in bed attempting further sleep until the lights were switched on. Loudspeakers were present in all bedrooms, irrespective of whether used or not.

#### Study schedule

Fig. 1 shows the study visit schedule. Subjects arrived fasted on Friday morning for blood sampling (glucose, insulin, c‐peptide, leptin, peptide YY (PYY), total ghrelin, cortisol, total testosterone and luteinizing hormone (LH)), verification of their sleep compliance with actigraphy data and to answer the Epworth Sleepiness Score (ESS)^13^ and Horne‐Ostberg Morningness‐Eveningness Questionnaire (MEQ).^14^ Height and weight were measured by standard methods. Subjects were asked to refrain from caffeine completely from that time. They returned to the facility on Friday from 5 pm and then did not leave the facility until after tests were completed on Monday morning. Following each night of the study condition, fasting blood samples were taken immediately after wake up (for glucose, insulin, c‐peptide, leptin, PYY, total ghrelin, cortisol, testosterone and LH). On Monday morning within 30–60 min of wake up, subjects underwent a frequently sampled (19 samples), two‐hour oral glucose tolerance test to determine insulin sensitivity. After baseline fasting hormone levels were taken through an intravenous cannula, 75 g of glucose was administered orally, then samples were taken after 5, 10, 15, 20, 25, 30, 35, 40, 45, 50, 55, 60, 70, 80, 90, 100, 110 and 120 min for insulin, c‐peptide and glucose measurements. Samples were centrifuged immediately and frozen to −80 °C until assayed. Detailed hormonal assay methodology can be found in Table S1. Insulin sensitivity was determined by minimal model analysis.^15^, ^16^ Area under the curve (AUC) for glucose and insulin was calculated using the trapezoid rule. HOMA‐IR, HOMA‐β^17^ and QUICKI^18^ indices of insulin sensitivity were calculated.

#### Polysomnography and slow‐wave sleep suppression

Polysomnography was recorded each night using standard electrode placement (Sandman Elite V.9.2, Tyco Healthcare, Denver, Colorado, USA). Leads were referenced to the contralateral mastoid position. Sleep stages were scored using standardized criteria^19^ by one scorer, with strict attention to delta‐wave voltage criteria. SWS was suppressed using acoustic stimuli on all 3 weekend nights of Condition C. Delta waves were recognized visually in real‐time on the central leads of the electroencephalogram (EEG) by the researchers. When two or more consecutive delta waves were seen, a mixed frequency ramped tone was played through bilateral loudspeakers next to the subject's bed, ramping from 40 to 95 dB (measured at the approximate location of the subject's head), until delta activity was suppressed. If the maximum volume tone did not control delta activity, the researchers would go into the bedroom, gently disturb the subject and say their name.

#### Power spectral analysis

Power spectral analysis was performed on a central lead of the EEG to determine non‐rapid eye movement (NREM) mean delta power, NREM relative delta power density (% delta power/total power across all frequency bands) and total NREM delta power (mean delta power x number of 30 s epochs ×2) after removal of EEG artefact using an automated method with visual verification.^20^ If noise artefact was present in over 25% of the channel, it was discarded from analysis (10/114 studies). Lead C3‐M2 was utilized unless the signal quality was suboptimal, whereby C4‐M1 was substituted for all six nights for that subject (*n* = 4). Fast Fourier transformation was performed on 5 s epochs over the entire frequency bands, with the delta range (0·75–4·5 Hz) the primary focus for analysis.^20^

#### Food intake and exercise

Meals were chosen from a menu, which included healthy balanced frozen meals for breakfast, lunch and dinner, with snacks available. Quantity of food was not restricted over the 1st weekend visit. During the 2nd weekend study visit, each subject was served exactly the same meals and snacks they had consumed during the first weekend, to ensure dietary intake was standardized over both weekends. Food intake for each individual was summed from the available nutritional information. No caffeine, alcohol or chocolate was available. Breakfast was served 30 min after the subject's wake‐up time, lunch at 12·30 pm and dinner at 6·30 pm. All subjects obliged with the dietary instructions and minimal deviation occurred, except occasionally for food availability, when a similar meal was provided. Diet was not monitored in between study visits.

### Statistical analysis

zOur primary aim was to determine whether ‘catch‐up’ sleep would improve insulin sensitivity, and our exploratory aim was to unravel potential mechanisms by which this might occur, such as through changes in SWS and/or hormones known to be metabolically active. The primary outcome was the difference in insulin sensitivity, determined by minimal modelling, after three nights of each sleep condition. Secondary outcomes were disposition index and hormones (leptin, PYY, ghrelin, cortisol, testosterone, LH). Tertiary outcomes were insulin sensitivity measured by HOMA and QUICKI, fasting and/or AUC glucose, insulin and c‐peptide. The polysomnographic findings are not outcomes – these variables were analysed to verify that the intervention (i.e. ‘catch‐up’ sleep, SWS suppression, Cary) altered sleep duration and architecture as expected. Data were analysed using SAS version 9.2 (SAS Institute, Cary, North Carolina, USA) using paired t‐tests and mixed model analysis for repeated measures where appropriate incorporating ‘condition’, ‘day’ and interaction terms, with two‐tailed *P* values <0·05 considered significant. Normality of data or of residuals was assessed, as appropriate. Data transformation was not required. Period and crossover effects were excluded from available baseline data of each weekend.^21^ Results were assessed separately for group 1 compared to overall, and a ‘group*condition’ term was utilized to assess for any age interaction of the older group on the overall results. Data are described as means and standard errors, or differences and 95% confidence intervals as appropriate.

## Results

### Demographics

315 people responded to advertising; 49 attended full screening, of which 21 men were randomized: 18 in group 1 (≤35 years) and three in group 2 (>35 years), with 19 subjects completing both weekend visits. The main reasons for screen failures were not exhibiting sufficient sleep restriction during the working week (*n* = 9 of 28; 32%), or not reaching the criteria set of 25% ‘catch‐up’ sleep on weekends (*n* = 6 of 28; 22%). In group 1, one subject was randomized who did not undergo either weekend visit and another subject withdrew following 1 weekend due to needle phobia. Due to within‐person study design, neither individual could be analysed. The following participants completed each of the three possible condition pairings:

10 h TIB/6 h TIB: *n* = 8
10 h TIB/10 h↓SWS: *n* = 6
6 h TIB/10 h↓SWS TIB: *n* = 5.

Screening characteristics are shown in Table 1, demonstrating subjects were sleep restricted during the working work (6 h 12 min/night ± 7 min). All men showed a significant increase in weekend sleep compared to weekday sleep (mean weekend sleep extension 37·3% ± 2·4) (Fig. S1). Hence, a 6‐h sleep opportunity was almost identical to the average time spent asleep during weekdays, whereas a 10‐h sleep opportunity exceeded the time each slept during weekends (Fig. S1). All subjects had habituated to these sleep patterns regularly at home for at least 6 months and on average 5·1 years ± 0·9. The most common reason for these sleep patterns was working long hours, alongside studying and time commuting to and from work and/or study. MEQ excluded preference for morning or evening (mean 47·3 ± 1·5; ‘neither type’ category range 42–58^14^). Other than age and BMI being higher, descriptively the older group did not alter the overall mean demographics. ESS was within the normal range, excluding subjective sleepiness. No significant differences in BMI or sleep parameters by actigraphy for the 2 weeks leading up to study visits were found between the two weekends (Table S2).

**Table 1:** Screening characteristics (means ± SEM)

*n* = 19	Mean ± SEM	Range
Age (years)	28·6 ± 2·0	19–49
Midweek sleep^a^	6 h 12 min ± 7 min	5 h 18 min–6 h 54 min
Weekend sleep^b^	8 h 30 min ± 9 min	6 h 59 min –9 h 39 min
Weekend sleep extension^c^ (%)	37·3 ± 2·4	19–56
Duration of catch‐up sleep patterns (years)	5·1 ± 0·9	0·5–15
MEQ score^d^	47·1 ± 1·5	34–58

a Defined as average rest period Monday to Thursday inclusive over 2 weeks of screening by actigraphy and diaries.

b Defined as average rest period Friday and Saturday over 2 weeks of screening.

c Defined as % more weekend sleep compared to midweek sleep over 2 weeks of screening.

d MEQ‐ Horne‐Ostberg Morningness‐Eveningness Questionnaire (definite evening type 16–30; moderate evening type 31–41; neither type 42–58; moderate morning type 59–69; definite morning type 70–86).

### Sleep parameters – the intervention

**Figure 2:**
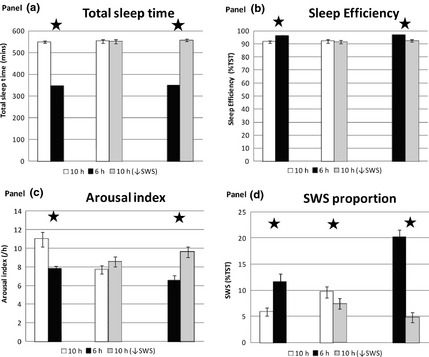
Polysomnographic sleep parameters between pairs of conditions averaged over three experimental nights. (a) TST (mins), (b) sleep efficiency (%TST), (c) arousal index (events/h), (d) SWS proportion (%TST) 10 h/6 h *n* = 8, 10 h/10 h↓SWS *n* = 6, 6 h/10 h↓SWS *n* = 5. Error bars are SEM. * represents significance *P* < 0·05.

PSG sleep parameters and power spectral analysis results are shown in Figs 2 and Fig. S2. Across the pairs of conditions, expected significant differences were seen in total sleep time (TST) (Fig. 2a), sleep efficiency (percentage time asleep during time in bed) (Fig. 2b) and sleep latency (Fig. 2a). Notably, sleep efficiency exceeded 90% for all conditions, and the 10 h↓SWS condition compared to 10 h did not significantly reduce TST nor sleep efficiency, despite the acoustic stimuli (Fig. 2a,b). The 6h condition had a significantly reduced arousal index compared to 10 h (*P* < 0·001) or 10 h↓SWS (*P* < 0·001), consistent with maintaining a more consolidated sleep with sustained sleep restriction (Fig. 2c). Arousal index in the 10 h↓SWS condition compared to 10 h, although higher, did not reach significance (*P* = 0·09). The 10 h↓SWS condition reduced SWS quantity by 23% (−12·6 min, (−23·4, −1·8); *P* = 0·02) compared to 10 h and by 62% compared to 6 h (−43·6 min, (−55·0, −32·36); *P* < 0·001), as expected by the experimental protocol (Fig. S2b). The 6h condition had the highest SWS proportion (%TST) across all pairs of conditions (compared to 10 h, *P* < 0·001; compared to 10 h↓SWS, *P* < 0·001) (Fig. 2d).

In examining the delta power of the EEG, 10 h↓SWS reduced mean NREM delta power by 10% (−41·7 μV^2^, (−69·3, −13·9); *P* = 0·005) and relative delta power compared to 10 h (*P* = 0·0002), as anticipated by the acoustic stimuli protocol (Fig. S2e,f). The 6‐h condition had significantly higher mean NREM delta power and relative delta power compared to either 10 h (*P* < 0·001) or 10 h↓SWS (*P* < 0·001), as expected with sustained sleep restriction (Fig. S2e,f).

### Metabolic outcomes‐ insulin sensitivity

**Figure 3:**
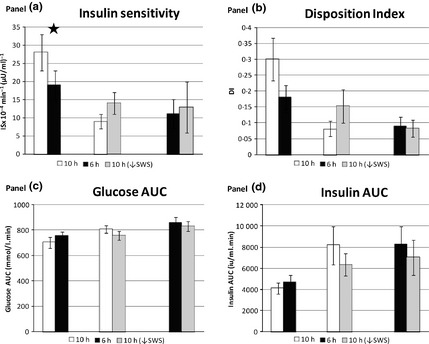
Metabolic outcomes between pairs of conditions from minimal model of an oral glucose tolerance test performed on Monday after three nights of each condition. (a) insulin sensitivity (/min/(μU/ml)), (b) disposition index, (c) glucose area under the curve (AUC‐ mmol/l. min), (d) insulin AUC (iu/ml. min). 10 h/6 h *n* = 8, 10 h/10 h↓SWS *n* = 6, 6 h/10 h↓SWS *n* = 5. Error bars are SEM. * represents significance *P* < 0·05.

**Figure 4:**
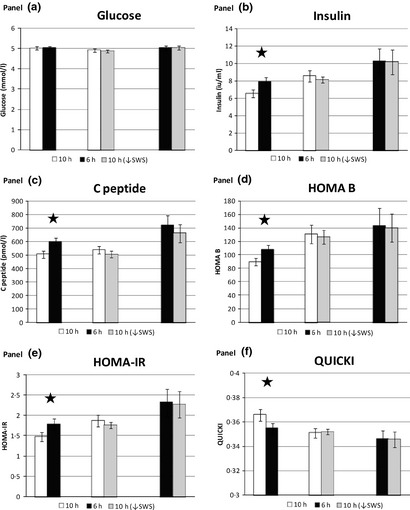
Metabolic outcomes between pairs of conditions from daily fasting blood samples showing mean values across Sat/Sun/Mon. (a) glucose (mmol/l), (b) insulin (iu/ml), (c) c‐peptide (pmol/l), (d) HOMA‐β, (e) HOMA‐IR, (f) QUICKI. 10 h/6 h *n* = 8, 10 h/10 h↓SWS *n* = 6, 6 h/10 h↓SWS *n* = 5. Error bars are SEM.* represents significance *P* < 0·05.

Results for the main metabolic parameters are shown in Figs 3 and 4. Period and carryover effects were excluded by analysing Friday baseline values where available. Insulin sensitivity (ISx) was significantly increased following three nights of ‘catch‐up’ sleep (10 h) compared to continuing sleep restriction (6 h) (8·57 × 10^−4^/min/(μU/ml), (1·1, 16·1 × 10^−4^; *P* = 0·03) (Fig. 3a). There were no significant differences between 10 h↓SWS and either 10 h (*P* = 0·17) or 6 h (*P* = 0·6). Changes of similar magnitude and direction were seen for disposition index (DI), but these were not statistically significant (Fig. 3b). Glucose AUC was significantly lower in 10 h compared to 6 h in the younger men −69·2 mmol min.L^−1^ (−119·7, −18·6); *P* = 0·02), but not in the young and old men together (*P* = 0·14) (Fig. 3c). Insulin AUC differences were not significant (Fig. 3d). Daily fasting morning hormone levels showed significant reductions in fasting insulin, c‐peptide, HOMA‐IR and HOMA‐β and an increase in QUICKI following 10 h compared to 6 h (Fig. 4) – all consistent with improvements. Only 1% of insulin, c‐peptide and glucose values were missing. Certain results showed an age effect, with the older subjects having higher C‐peptide, glucose and leptin levels; however, this did not alter the overall significances of differences when an age factor was applied to the model.

### Metabolic outcomes – appetite hormones, cortisol and testosterone

**Figure 5:**
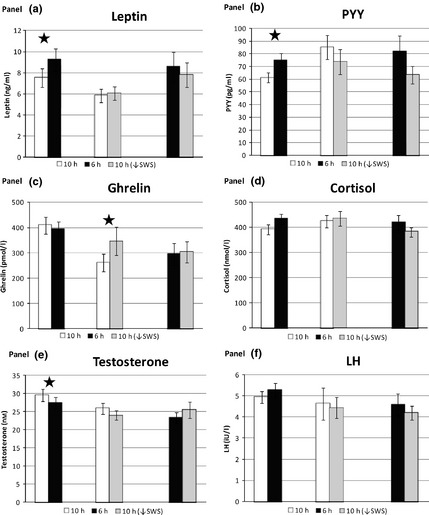
Metabolic outcomes between pairs of conditions from daily fasting blood samples showing mean values across Sat/Sun/Mon. (a) leptin (ng/ml), (b) PYY (pg/ml), (c) ghrelin (pmol/l), (d) cortisol (nmol/l), (e) testosterone (nm), (f) lutenizing hormone (LH‐iU/l). 10 h/6 h *n* = 8, 10 h/10 h↓SWS *n* = 6, 6 h/10 h↓SWS *n* = 5. Error bars are SEM. * represents significance *P* < 0·05.

Leptin was significantly reduced following 10h ‘catch‐up’ sleep compared to 6 h (−1·69 ng/ml (−0·6, −2·8); *P* = 0·003), along with a corresponding reduction in PYY (−12·7 pg/ml (−2·1, −23·3); *P* = 0·02), but no change was seen in total ghrelin (*P* = 0·59) (Fig. 5a–c). There was no significant change in fasting morning cortisol levels between any of the condition pairings (Fig. 5d).

The amount of food consumed between weekend visits was not significantly different for each individual (1st weekend = 6230 kcal, 2nd weekend = 6291 kcal; *P* = n/s). Nor was there any significant difference between the amount of energy intake between sleep conditions, when specifically looking at only the first weekend chronologically when food choices were made, independent of condition pairing (10 h = 6394 kcal, 6 h = 5845 kcal, 10 h↓SWS = 6426 kcal; *P* = n/s). Only food choices from the first weekend were analysed because subjects were not allowed to rechoose on the second weekend. Furthermore, when exploring only those in the 10 h/6 h condition pairing, no significant difference was seen between food choice as determined by energy intake on the first weekend (10 h = 6250 kcal, 6 h = 5844 kcal; *P* = n/s). Fasting morning testosterone levels were significantly higher following 10 h compared to 6 h (2·2 nm (0·2, 4·2); *P* = 0·03) in both the group as a whole (*n* = 8) and in the younger group alone (*n* = 5) (Fig. 5e). The older men (*n* = 3) had lower levels compared to the younger men, as expected with ageing (*P* = 0·01). LH was not significantly different between any of the condition pairings (Fig. 5f).

## Discussion

‘Catch‐up’ sleep is highly prevalent with >40% of working aged adults sleeping more on weekends compared to weekdays.^2^ Understanding the metabolic implications of these lifestyle choices is therefore highly relevant. We show that men who regularly adopt lifestyle‐driven, chronic, repetitive sleep restriction with weekend ‘catch‐up’ sleep significantly improved insulin sensitivity by 45% following three nights of a saturating sleep compared to ongoing sleep restriction, as measured by minimal model after an oral glucose challenge. HOMA‐IR decreased and QUICKI increased. Accordingly, three separate measures of ISx all showed improvements with ‘catch‐up’ sleep. These data are novel and together attest to the veracity of this finding. Previous studies have shown that sleep restriction of one night to 2 weeks has a negative impact on markers of glucose homoeostasis,^22^, ^23^, ^24^, ^25^ but have examined subjects with regular sleep patterns, unlike those in our study. Our finding of a 45% improvement in ISx with ‘catch‐up’ sleep is complementary and consistent with previous studies showing a 20–25% worsening of ISx with sleep restriction.^1^

‘Catch‐up’ sleep decreased fasting insulin, c‐peptide and HOMA‐β, likely reflecting the concomitant improvement in ISx. ‘Catch‐up’ sleep increased morning testosterone and did not change morning cortisol. These findings are consistent with other studies of sleep restriction.^26^, ^27^ Randomized controlled trials directly show that testosterone treatment improves ISx in men who are obese,^28^ as well as in men with disrupted and reduced sleep from obstructive sleep apnoea.^29^ Testosterone improves glycaemic control in men with type 2 diabetes mellitus^30^ and reduces obesity and metabolic syndrome.^31^ Meta‐analyses show significant reductions in fasting plasma glucose, fat mass and triglycerides with testosterone therapy in men with type 2 diabetes mellitus.^30^ Previous studies have shown that sleep restriction can increase evening, but not morning, cortisol,^22^, ^24^, ^25^, ^26^ with no change in mean cortisol across 24 h.^1^ Interventional studies conclusively show that increased afternoon/evening cortisol worsens insulin resistance in humans^32^ and rodents.^33^ These findings occur because maintaining cortisol concentrations during the 4–6 h of the circadian nadir (early evening) is important to avoid effects of glucocorticoid excess on peripheral tissues.^33^ Whether or not sleep impacts insulin sensitivity through these hormonal changes is plausible, but remains to be determined.

We examined satiety and hunger hormones released by adipose tissue (leptin‐satiety signal), small intestine (PYY‐satiety) and stomach (ghrelin‐hunger) as secondary outcomes. ‘Catch‐up’ sleep decreased leptin and PYY compared with continued sleep restriction, but did not alter ghrelin or food choice determined by energy intake. Studies have shown conflicting changes in appetite hormones with sleep restriction due to differing food intake, energy balance at time of assessment, gender differences and possible changes in circadian rhythm.^1^, ^26^, ^34^ However, our subjects ate the same meals across both weekends, albeit *ad libitum* during the first weekend. Energy expenditure was not measured; however, exercise was not allowed. Circadian shift was minimized, as sleep opportunity was centred individually to home sleep patterns and lighting was <50 lux. Although decreased leptin and PYY should decrease satiety, we did not observe a change in energy intake. Although surprising, these data are consistent with recent data showing sleep restriction increased leptin and PYY, and decreased ghrelin in a carefully conducted study utilizing 24‐h assessment of these hormones^34^. We found no change in ghrelin with ‘catch‐up’ sleep, although decreasing SWS increased ghrelin. This novel finding requires replication in other studies, as we did not adjust for multiple testing for this or any of the other secondary outcomes.

Slow‐wave sleep is a metabolically active sleep stage, and others have shown that disrupting SWS can worsen ISx.^11^ In our hands, targeted acoustic stimuli significantly disrupted SWS and reduced delta power, but the absolute effect, although significant, was small in magnitude. ISx was not altered, in contrast with previous studies.^11^, ^35^ This discrepancy could be explained if a minimal reduction in SWS required to worsen ISx was not achieved in our chronically sleep‐restricted subjects, or if other factors such as sleep fragmentation and/or arousals *per se* are ultimately responsible^35^. On the other hand, our study was likely underpowered to show an effect of SWS suppression on ISx, in part because both baseline SWS and ability to suppress SWS were highly variable in our study population and also because it proved to be much more difficult to suppress SWS in a population that is chronically sleep deprived than we had originally anticipated.

These experimental findings exploring chronic repetitive sleep restriction are highly relevant because such sleep patterns are common in modern society and it has been suggested that chronic sleep restriction leads to the development of obesity and diabetes mellitus,^36^ in addition to other cardiometabolic consequences.^37^ Over a prolonged period of time (years or decades), this improvement in insulin sensitivity could be highly relevant in delaying or even preventing prediabetes or type 2 diabetes mellitus in a relatively healthy young individual. In a population of millions of individuals, this change in insulin sensitivity would translate to decreased prediabetes and diabetes mellitus in the community. Furthermore, interventional studies now show that sleep restriction increases weight^38^ and decreases fat proportion lost in those trying to lose weight through planned negative energy balance.^1^ Studies attempting to manipulate sleep in the home setting have not been adequately powered to show changes in ISx given the increased variability that can occur in an uncontrolled non‐laboratory environment. Nevertheless, larger community‐based sleep extension trials are required, but need to be sufficiently large to account for variable adherence to the sleep intervention, the introduction of confounders outside of the laboratory and possibly for a between‐group study design.

Indeed, this wide variability in ISx is one potential limitation for our investigation. This variability was readily observed by examining the interindividual differences in response to 10 h of sleep repletion (Fig. 3a) and could be related to age, lifetime duration of chronic sleep deprivation, degree of at‐home sleep restriction or many other variables. In fact, these factors may contribute to the wide variability observed in ISx in the general population. Our sample size was too small for us to determine these factors, but the goal of the study was to determine effects of recovery ‘catch‐up’ sleep on ISx, and here, the crossover study design allowed a paired statistical analysis to examine the effect of sleep repletion within the same person, using 57 measurements (19 measurements each for insulin, C‐peptide and glucose) to precisely measure ISx, thereby negating the impact of interindividual differences in ISx among individuals. Indeed, paired Student's *t*‐tests, as we implemented, remain valid without an increase in type 1 error over 0·05 even with these sample sizes^39^ and Student's original paper utilized a sample size of 4.^40^ Another possible limitation is that 3, not 2, nights of ‘catch‐up’ sleep was tested, whereas the latter might be more consistent with a weekday/weekend pattern. However, our proof‐of‐concept study of three nights ‘catch‐up’ sleep is still feasible in the community, wherein additional sleep on the 3rd (Sunday) night could be achieved with an earlier bedtime. Nevertheless, further studies of one and two nights of sleep repletion are needed to explore the chronology of metabolic recovery. Our population was specifically in individuals with ‘catch‐up’ sleep patterns and may not be generalizable to other populations including those with other sleep disorders, such as obstructive sleep apnoea.

Our study examines, for the first time, a population regularly using ‘catch‐up’ sleep. We show that ‘catch‐up’ sleep improved ISx over continued sleep restriction, thereby confirming that extending sleep is potentially beneficial at least in nondiabetic men with long‐standing chronic, repetitive sleep restriction. Critically, our intervention of 10‐h sleep opportunity translated to actual sleep as sleep efficiencies >90% and exceeded the usual amount of sleep extension of every participant, raising the possibility that their habitual attempts at ‘catch‐up’ sleep were suboptimal. These data suggest that many in our society should sleep more, but further studies will be required to determine how much more sleep is needed in which specific individuals and whether planning to consistently sleep more every night is, in the long‐run, ultimately superior to the occasional 1, 2 or 3 nights of ‘catch‐up’ sleep.

## Supporting information


**Figure S1** Screening sleep period times – weekend sleep period (mean min/night of Fri/Sat inclusive) *vs* midweek (mean min/night Mon to Thurs inclusive).
**Figure S2** Additional polysomnographic sleep parameters and power spectral analysis results between pairs of conditions averaged over 3 experimental nights.
**Table S1:** Methodology‐ Detailed Hormonal Assay Information
**Table S2:** Baseline characteristics between study visits
