# Supplementary material for: Metabolic and hormonal effects of ‘catch‐up’ sleep in men with chronic, repetitive, lifestyle‐driven sleep restriction
Source: Clin Endocrinol (Oxf). 2015 Mar 6;83(4):498–507. doi: 10.1111/cen.12747 (PMC4858168; doi:10.1111/cen.12747)
Supplement: Supplementary file 1 — Figure S1 Screening sleep period times – weekend sleep period (mean min/night of Fri/Sat inclusive) vs midweek (mean min/night Mon to Thurs inclusive). Figure S2 Additional polysomnographic sleep parameters and power spectral analysis results between pairs of conditions averaged over 3 experimental nights. Table S1: Methodology‐ Detailed Hormonal Assay Information Table S2: Baseline characteristics between study visits [file CEN-83-498-s001.docx]

**Supplementary Figure S1: Screening sleep period times**

Open triangles indicate those in the 10h/6h conditions (n=8). Closed symbols indicate those in either 10h/10h↓SWS or 6h/10h↓SWS conditions (n=11).

**
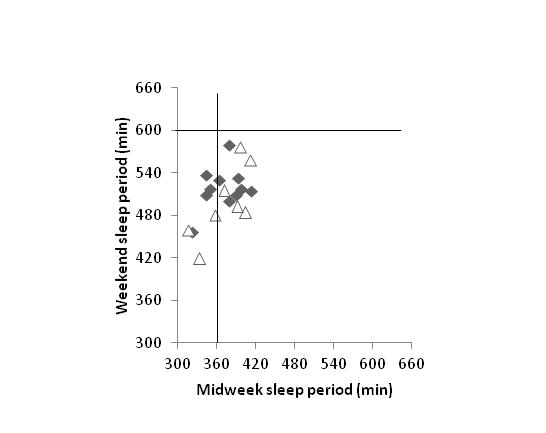
**

**Supplementary Figure S2: Additional polysomnographic sleep parameters and power spectral analysis results**

**
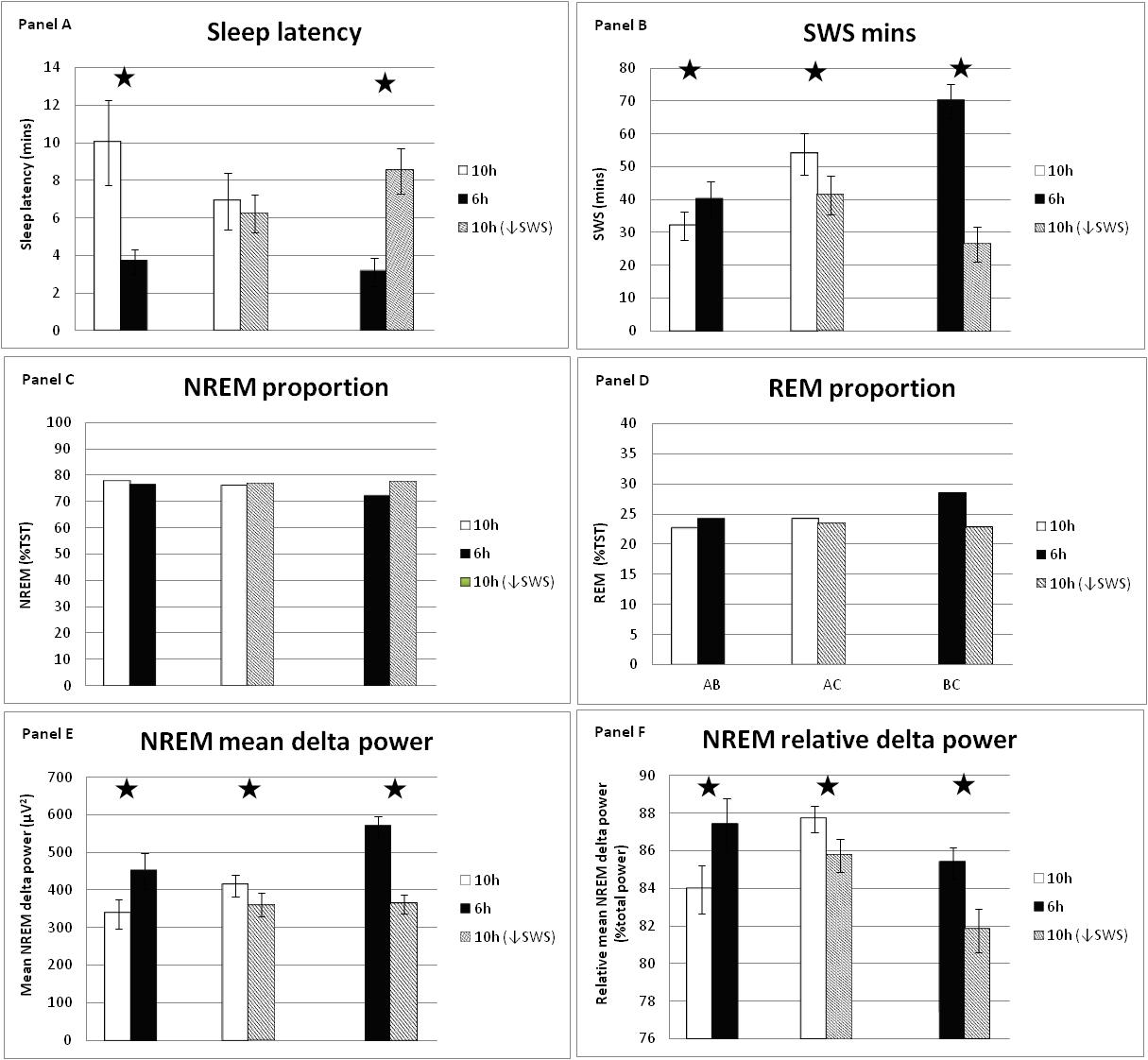
**

**Supplementary Table S1: Methodology- Detailed Hormonal Assay Information**

| **Test** | **Assay type** | **LLD** | **Inter-assay CV** | **Intra-assay CV** |
| --- | --- | --- | --- | --- |
| Glucose | Hexokinase^1^ | 0.1 mmol/L | <0.9% | <1.8% |
| Insulin | Standard platform assay^2^ | 1.0 uU/mL | <4.2% | <5.2% |
| C-peptide | Standard platform assay^3^ | 99pmol/L | ≤15% | ≤15% |
| Leptin | Radioimmunoassay^4^ | 0.437 ng/ml | ≤8.3% | ≤6.2% |
| PYY | Radioimmunoassay^4^ | 8 pg/mL | ≤6.75% | ≤4.66% |
| Ghrelin | Radioimmunoassay^4^ | 93 pg/mL | ≤10% | ≤16.7& |
| Cortisol | chemiluminescent enzyme immunoassay^3^ | 5.5nmol/L | ≤7.5% | ≤9.5% |
| Testosterone | Delfia assay^5^ | 0.1nM | 9.1% | 3.1% |
| LH | Delfia assay^5^ | 0.02IU/L | 8.6% | 4.2% |

LLD- lower limit of detection; CV- coefficient of variation; n/a- not available

^1^ Modular P^TM^, Roche Diagnostics, Castle Hill, NSW, Australia

^2^ Architect^TM^, Abbott Laboratories, Abbott Park, IL, USA

^3^ Immulite^TM^ 2000, Diagnostic Products Corporation, Los Angeles, CA, USA

^4^ Merck Millipore, Merck Germany, Germany

^5^ Perkin-Elmer Life Sciences, Rowville, Australia

**Supplementary Table S2: Baseline characteristics between study visits (means±SEM; p value by student’s t-test)**

| **n=19** | **Weekend 1** | **Weekend 2** | **p value** |
| --- | --- | --- | --- |
| Weight (kg) | 83.7±3.6 | 83.7±3.6 | 0.85 |
| BMI (kg/m^2^) | 26.0±0.8 | 26.0±0.9 | 0.88 |
| Midweek sleep prior to visit^1^ | 6h 6m±8m | 5h 59m±8m | 0.32 |
| Weekend sleep prior to visit^2^ | 8h 34m±12m | 8h 34m±14m | 0.98 |
| % weekend extension sleep^3^ | 41.2±4.1 | 43.6±3.0 | 0.52 |
| ESS (/24) | 5.0±0.7 | 5.5±0.7 | 0.54 |

^1^Defined as average rest period Monday to Thursday inclusive over 2 weeks by actigraphy and diaries

^2^Defined as average rest period Friday and Saturday over 2 weeks

^3^Defined as % more weekend sleep compared to midweek sleep over 2 weeks

BMI- body mass index, ESS- Epworth Sleepiness Scale
